# Assessing Canine Parvovirus Vaccine Performance in Puppies with Maternally Derived Antibody: An Improved Study Design

**DOI:** 10.3390/vaccines13080832

**Published:** 2025-08-04

**Authors:** Jacqueline Pearce, Ellen Versmissen, David Sutton, Qi Cao, Ian Tarpey

**Affiliations:** 1 MSD Animal Health, 5831 Boxmeer, The Netherlands; ellen.versmissen@merck.com (E.V.); qi.cao2@merck.com (Q.C.); ian.tarpey@msd.com (I.T.); 2 MSD Animal Health, Milton Keynes MK7 7AJ, UK; david.sutton@msd.com

**Keywords:** canine parvovirus, maternal immunity, vaccination, vaccine efficacy, puppies

## Abstract

**Background/Objectives**: Typically, studies aiming to assess the ability of canine parvovirus (CPV) vaccines to immunise puppies with maternally derived antibody (MDA) are undertaken using group-housed puppies. Since live attenuated vaccine virus is invariably shed in the faeces, this can result in repeated oral re-exposure and puppies which failed to respond to the initial vaccination may respond instead to shed vaccine virus in the environment, thus artificially enhancing the efficacy of the vaccine. This problem can be avoided by adopting a pair-housed study design where one vaccinated pup is housed with one unvaccinated sentinel. Using this design, we examine the capability of four commercially available canine parvovirus vaccines to immunise MDA-positive pups. **Methods**: Thirty-four 6-week-old puppies born to vaccinated dams were divided into four vaccine groups with similar MDA ranges. Within each group puppies were paired based on matching MDA titres, and each pair was housed in separate biocontainment accommodation. In each pair, the pup with the highest MDA was vaccinated and the other left as an unvaccinated sentinel. All vaccinates were given a single dose of one of the vaccines. Vaccinates and sentinels were then bled every 2–4 days and CPV antibody was measured. Daily rectal swabs were also collected from all pups to identify any shed vaccinal CPV. **Results**: All the pups vaccinated with Nobivac DP PLUS seroconverted, with significantly higher antibody titres compared to the pups in other vaccine groups, all shed vaccine virus, and all bar one of the sentinel pups seroconverted. In the other groups, only vaccinated pups with lower levels of MDA seroconverted and shed vaccine virus but none of the sentinel pups seroconverted. **Conclusions**: Different canine parvovirus vaccines differ in their ability to replicate in and immunise puppies with MDA, the levels of which may vary widely between individuals. The shedding of vaccinal CPV is an important consideration when designing studies to demonstrate efficacy in MDA-positive puppies.

## 1. Introduction

Maternally Derived Antibody (MDA) is antibody, predominantly of the IgG isotype, that is transferred from mother to neonate to provide the neonate with a temporary level of passive immunity whilst the immature immune system develops [1]. MDA transfer in the dog occurs primarily through the absorption of colostrum across the gut, but the opportunity for such transfer is limited. At birth, the gut is permeable to macromolecules such as immunoglobulins which can be absorbed undigested into the neonatal circulation. However, gut closure begins very swiftly by 4 h after birth and is complete by 16–24 h, a phenomenon which appears to occur earlier in puppies than in many other species [2]. Circulating MDA levels in puppies, even within a single litter, can differ markedly depending on their access to colostrum and the immune status of the mother [3,4]. Although MDA plays an important and beneficial role in protecting young puppies against disease, it is only present for a relatively short period. Since it is passive antibody, MDA titres decline over time with a half-life ranging from 8 to 14 days, providing protection for up to three to four months depending on the disease in question and the initial level of MDA [4,5,6]. Notwithstanding the essential role MDA plays in neonatal disease prevention, it has been recognised for many decades that circulating MDA, if present in sufficient quantity, can neutralise vaccine antigens and prevent successful immunisation [6,7,8,9,10,11].

Canine Parvovirus Type-2 (CPV-2) is a major viral pathogen of dogs that can cause severe acute gastroenteritis associated with a high morbidity and mortality, particularly in young puppies [12,13]. It is nowadays recognised as one of the most common infectious diseases of dogs worldwide [12]. As a result, vaccination against the disease is universally defined as ‘core’ by all independent expert vaccine guideline groups, meaning that all dogs, especially young puppies, irrespective of geography or lifestyle, should be routinely vaccinated [6,14]. Although the currently available modified live CPV-2 vaccines are highly effective, interference with CPV vaccination by MDA is well recognised [9,10,15] and has been described as one of the main causes of immunisation failure [15]. As a consequence, vaccine manufacturers have been attempting for some time to develop vaccines and vaccination strategies to overcome this problem. The strategies largely involve repeated vaccination from 4–6 weeks of age onwards to immunise when the MDA level is low enough for the vaccine to take without leaving the animal exposed to risk of field infection for too long [6,14]. Additionally, many improved CPV-2 vaccines have been developed over the years which immunise more effectively in the face of MDA [16,17,18,19,20].

It is important that studies designed to assess the ability of CPV vaccines to immunise in the face of MDA produce accurate and reliable results; however, one aspect of the design can potentially lead to the performance of the vaccine being overestimated. CPV is a small, non-enveloped DNA virus that is resistant to desiccation and can be difficult to remove from animal house environments [12]; this is particularly relevant since most current CPV vaccines are live attenuated strains which replicate in the gut and will commonly be shed in the faeces [21,22,23]. Once in the environment, CPV can readily persist and infect other puppies in the group. The vaccine strains can all potentially immunise following ingestion, meaning that any puppies which initially failed to respond to the vaccine dose because of MDA interference may nonetheless respond to later oronasal exposure from shed vaccine virus once their MDA has declined to non-interfering levels. In these group-housed studies, especially where serological monitoring is infrequent, it is practically impossible to distinguish between a puppy which responded to the administered vaccine versus one which responded to later environmental exposure from shed vaccine strain.

In this paper, we describe an improved study design which eliminates this possibility and provides a more precise measure of vaccine performance. Using this design, we examine the serological response of MDA-positive puppies following vaccination with several commercially available CPV vaccines, including one containing a recently approved novel recombinant strain which has been shown to immunise in the face of high levels of MDA [20]. Furthermore, the re-isolation of vaccine virus and subsequent spread to a co-housed sentinel animal for each of the CPV vaccines is investigated.

## 2. Materials and Methods

### 2.1. Animals and Housing

Studies were performed in 4–7-week-old beagle pups born to vaccinated SPF mothers, obtained from a registered UK supplier. All animals were fed a standard commercial dog diet with water available *ad libitum*. In order to ensure that all pups thrived equally, the provision of puppy supplements, milk replacements, and treats was permissible and was at the discretion of the animal care staff.

Animals were housed under conditions of strict biocontainment. Pairs of dogs in any one study group were housed in accommodation which was separately biocontained from other pairs. Full biocontainment measures were also in place to ensure no transfer of virus between different groups.

### 2.2. Ethics Statement

These studies were performed in compliance with Directive 2010/63/EU on the protection of animals used for scientific purposes as transposed into the national law of the applicable country. The animal studies were approved by the MSD Animal Health Animal Welfare Body and were performed in compliance with the UK Animals Scientific Procedures Act 1968 (license number P030CB685, approved on 22 August 2017). Prior to the start of each study each animal was rectally swabbed and shown to be negative for the presence of CPV on virus isolation, and each animal underwent a veterinary examination and was declared to be clinically healthy and suitable for inclusion. The animals were continuously monitored under veterinary care throughout the studies.

### 2.3. Vaccines

The commercially available vaccines used for vaccinating the MDA-positive puppies are shown in Table 1.

### 2.4. Blood Sampling and Serology

All dogs were bled from the cephalic vein. Clotted blood samples were taken into normal glass vials for serology and allowed to clot overnight at 2–8 °C or clotted for a few hours at room temperature followed by 2–8 °C. Clots were removed by centrifugation and the cleared sera stored below −15 °C until required for testing. All samples were analysed ‘blind’; they were labelled with an unrelated code which did not identify the puppy nor its MDA or vaccination status.

Serum samples were assayed for antibodies to canine parvovirus using haemagglutination inhibition [20,24,25] at a constant 4 HA units. Each serum sample was tested at two different dilutions against CPV-2a, CPV-2b and CPV-2c subtypes. The serum dilutions used depended on the predicted level of antibody present (for example, 1 in 2 and 1 in 5 for samples predicted to have a low titre, 1 in 4 and 1 in 5 for samples predicted to have a moderate/high titre). For most samples, the average of the results for both dilutions was calculated for each biotype. However, in the case of serum samples with antibody titres that were below (<) the limit of detection, only the HAI result from the lowest dilution is given. In the case of serum samples with antibody titres whose endpoint was above (>) the limit of detection, only the HAI result from the highest dilution is given. A validated positive reference serum and negative reference serum were included as assay controls.

In addition to measuring the three separate subtype (CPV-2a, CPV-2b, and CPV-2c) titres, a single ‘mean CPV titre’ for each puppy at each timepoint was calculated, this being the arithmetic mean of the three subtype titre values. This was done to obtain a single non-subtype-specific titre value to use when comparing the results between the different vaccine groups. The individual subtype titres can be seen in the Supplementary Data (Supplementary Table S1), and the mean titres are used in this paper (Table 2 and Table 3).

### 2.5. Virus Isolation from Rectal Swabs

Rectal swabs for virus isolation were taken into 1 mL of M6B8 tissue culture medium (MSD Animal Health) supplemented with 5% foetal bovine serum containing penicillin and streptomycin and were frozen until required for testing [26]. All samples were analysed ‘blind’; they were labelled with an unrelated code which did not identify the puppy nor its MDA or vaccination status. The swab suspension in each sample tube was defrosted, vortexed well, and centrifuged with the swab for 10 min at 1000× *g* at +4 °C to pellet gross debris. Each swab suspension was semi-quantitatively analysed in duplicate by 5-fold dilution in A72 or CRFK cells at the time of plating. The plates were incubated at 37 °C/5% CO_2_ for 3–4 days and analysed by CPV-specific immunofluorescence (IFA). The plates were fixed in methanol followed by staining with an anti-CPV IgG3 A2F8 (=Mab7) monoclonal antibody [27] and a goat anti-mouse FITC conjugate (SIGMA) [20].

### 2.6. Study Design

Thirty-four pups were selected from nine separate litters on the basis of their MDA levels against canine parvovirus and their general health. All puppies underwent a veterinary health examination prior to the start of the study; all were declared clinically healthy with no exclusions. Blood samples were taken on one or two occasions between 4 and 16 days prior to the planned vaccination date and CPV serology carried out. The pups were then divided into four vaccine groups of either 8 or 10 pups so that the pups in each group had similar ranges of MDA. Within each vaccine group pairs were assigned based, again, on their levels of MDA, so that the two paired pups had the most closely matching levels. Antibody levels between the pairs within each group ranged from low to high, representative of the equivalent levels found in pups of the same age in the field. The pup with the highest level of CPV antibody in each pair was the chosen vaccinate and the other the sentinel. Each pair was housed in a separate biocontainment room and therefore the non-vaccinated pup of each pair acted as a sentinel to enable detection, via seroconversion, of any vaccine virus excreted from its vaccinated partner. An overview of the groupings and the day 0 mean antibody titres is shown in Table 2. All vaccinated pups received a single vaccination with a dose of the respective vaccine on day 0.

All pups were bled on day 0 just prior to vaccination and then at intervals of 2–4 days thereafter. The blood samples were assayed for canine parvovirus serology to determine if and when seroconversion occurred. Daily rectal swabs were also taken from all pups to detect any shedding of live CPV.

**Table 2:** Pre-vaccination (day 0) mean HAI titres.

Group (Vaccine)	Vaccinate/Sentinel	Pup ID	Pup Sex	Mean HAI Titre Day 0
**1 (Nobivac DP PLUS)**	V1	8638	M	523
	S1	9012	F	469
	V2	8668	F	427
	S2	8644	F	427
	V3	9040	M	427
	S3	2681	F	117
	V4	8662	F	131
	S4	8654	M	93
	V5	8728	M	72
	S5	8648	M	47
**2 (Vanguard Plus 5)**	V1	1848	F	213
	S1	1990	M	120
	V2	9426	M	187
	S2	2318	F	93
	V3	9425	M	173
	S3	1988	M	87
	V4	1849	F	72
	S4	9429	F	60
**3 (Versican Plus P)**	V1	4602	M	480
	S1	4541	M	288
	V2	4603	F	395
	S2	4596	M	131
	V3	4546	F	288
	S3	4542	M	288
	V4	4545	M	131
	S4	4599	M	131
**4 (Eurican Primo P)**	V1	4601	F	480
	S1	4597	M	261
	V2	4547	M	373
	S2	4595	M	261
	V3	4544	F	261
	S3	4548	F	131
	V4	4543	M	131
	S4	4598	M	104

### 2.7. Statistical Analysis

As previously described, a single mean CPV titre was calculated for each pup at each sampling time. To statistically evaluate how these mean CPV titres differed between the different vaccine groups (1–4) over the whole study period, a mixed-effect ANOVA for repeated measures was used. In order to normalise the titre data, it was first log-transformed (log2) and then used as the response variable in the ANOVA. Vaccine group, study day, and group-by-day interaction were included as fixed effects, and whether the puppy was a vaccinate (V) or sentinel (S) was included as a random effect to adjust for intraclass correlation. A separate ANOVA for repeated measures analysis just using the vaccinated puppies (i.e., excluding the sentinels) was also undertaken using the log2-converted mean titres as the response variable and vaccine group, study day, and group-by-day interaction as fixed effects. For calculation purposes, titres that lay outside the detection limits were included with their closest whole number (e.g., <16 became 16). The level of significance was set at 0.05 and tests were two-sided. The SAS V9.4 (SAS Institute Inc., Cary, NC, USA) statistical software package was used.

## 3. Results

The mean CPV titres at each sampling time are shown in Table 2 and Table 3 and the individual subtype titres are available in the Supplementary Data (Supplementary Table S1). In all these tables, each of the vaccinate + sentinel pairs are numbered 1–4 or 5 in descending order based on the day 0 MDA titre of the vaccinate. Thus, the vaccinate with the highest day 0 titre is designated V1 and its paired sentinel S1. As can be seen in Table 3, the MDA titres in all pups initially decline as expected. Following successful seroconversion, the titres show a rapid and significant rise which is then maintained until the end of the study. The initial point of seroconversion is indicated by the start of the grey shading for the pup in question. All the Nobivac DP PLUS -vaccinated pups (Group 1) seroconverted sometime between 7 and 11 days post-vaccination, whereas from the other groups only two of the four vaccinates seroconverted, these being the pups with the lowest day 0 MDA titres.

**Table 3:** Serological response of the pups: mean haemagglutination inhibition titres (HAI units).

Group (Vaccine)	Pup ID	V/S *	Study Day										
			0	3	5	7	9	11	14	18	21	25	28
**1 (Nobivac DP PLUS)**	8638	V1	523	373	213	65	9216	11,947	23,893	23,211	20,480	11,947	11,947
	9012	S1	469	331	235	165	165	144	144	117	144	107	96
	8668	V2	427	373	187	87	800	7680	13,312	11,947	12,629	16,725	15,360
	8644	S2	427	373	288	235	240	165	17	13,653	25,259	20,139	9728
	9040	V3	427	334	288	144	144	13,392	>40,960	>40,960	>81,920	45,056	>40,960
	2681	S3	117	93	65	36	41	<16	96	9899	10,923	6997	4437
	8662	V4	131	83	36	939	9899	7253	16,725	18,432	12,629	17,067	9728
	8654	S4	93	53	53	47	36	72	8363	21,845	20,480	13,995	9728
	8728	V5	72	72	29	1600	11,947	11,947	20,651	18,432	13,995	17,067	10,581
	8648	S5	47	36	36	33	<16	3456	16,725	18,432	17,067	9216	7509
**2 (Vanguard Plus 5)**	1848	V1	213	144	144	131	93	144	117	65	41	36	30
	1990	S1	120	93	93	72	59	65	59	36	30	27	18
	9426	V2	187	131	133	107	131	261	144	72	59	41	36
	2318	S2	93	65	72	47	36	36	36	27	23	24	<16
	9425	V3	173	93	107	240	1877	2987	1877	1152	939	661	661
	1988	S3	87	72	72	53	36	33	36	27	23	21	17
	1849	V4	72	59	72	288	939	4608	3755	2645	2091	1877	2219
	9429	S4	60	53	41	29	30	33	24	18	18	18	<16
**3 (Versican Plus P)**	4602	V1	480	240	192	165	165	179	165	107	120	60	53
	4541	S1	288	165	144	165	131	131	107	85	79	53	53
	4603	V2	395	213	192	141	152	120	96	83	60	48	45
	4596	S2	131	83	72	77	65	53	53	36	30	18	21
	4546	V3	288	240	240	219	72	480	12,288	13,312	9216	7509	8533
	4542	S3	288	240	240	165	187	131	165	96	107	60	53
	4545	V4	131	83	83	83	288	9045	33,451	33,451	27,989	21,163	18,432
	4599	S4	131	65	59	53	60	47	60	33	30	18	19
**4 (Eurican Primo P)**	4601	V1	480	240	229	200	219	152	131	107	107	65	60
	4597	S1	261	131	120	107	120	76	83	52	41	33	35
	4547	V2	373	240	261	131	192	131	131	101	83	65	60
	4595	S2	261	131	131	107	120	107	83	65	60	33	41
	4544	V3	261	187	165	144	131	120	747	3072	2645	1493	1152
	4548	S3	131	76	83	60	53	47	48	33	30	18	17
	4543	V4	131	76	72	65	72	853	13,653	9728	6827	4181	4181
	4598	S4	104	60	53	67	38	30	48	33	30	18	18

* V = Vaccinate; S = Sentinel; Grey shading grey shading: Active seroconversion. The start of the grey shading is the first indication of an active immune response to vaccination.

The results of the statistical comparisons of the mean CPV antibody titres between the various vaccine groups are shown in Table 4. These support the findings shown in Table 3. The pups in Group 1 had significantly higher titres over all study days compared to all the other three groups. Excluding the sentinel pups from the analysis also shows that the pups vaccinated with Nobivac DP PLUS had significantly higher titres than pups vaccinated with any of the other vaccines.

**Table 4:** Statistical comparison of the mean CPV titres (log 2 converted) between Group 1 and the other groups (mean effect over all sampling days).

Parameter		Group Comparison	
	1 vs. 2	1 vs. 3	1 vs. 4
	*p*-Value, LS Mean (95% CI) *	*p*-Value, LS Mean (95% CI) *	*p*-Value, LS Mean (95% CI) *
Mean CPV titres (log 2 converted): all pups	<0.0001, 3.59 (2.24, 4.95)	0.0001, 2.65 (1.29, 4.00)	<0.0001, 3.15 (1.80, 4.50)
Mean CPV titres (log 2 converted): excluding sentinel pups	0.0112, 3.52 (0.95, 6.09)	0.0453, 2.63 (0.06, 5.20)	0.0164, 3.28 (0.70, 5.85)

* LS = Least Squares; CI = Confidence Interval.

The results of virus isolation assays from the daily rectal swabs revealed that shedding of live virus was detected in some but not all the vaccinated pups (Table 5 and Supplementary Table S2). All the pups vaccinated with Nobivac DP PLUS (Group 1) shed vaccine virus. Of the pups vaccinated with Versican Plus P (Group 3), both pups which seroconverted also shed virus, whereas of the pups vaccinated with Eurican Primo P (Group 4), shed virus was detected in only one of the two which seroconverted. Finally, shed virus was not detected in any of the pups vaccinated with Vanguard Plus 5. The groups also showed differences in respect of seroconversion and shedding of the sentinels. In Group 1, all the sentinels seroconverted, and all except one of the sentinels also shed virus. In Groups 2, 3, and 4, none of the sentinels seroconverted or shed virus. When interpreting the shedding data, it is important to bear in mind that only two cell-lines (A72 and CRFK) were used to try to isolate these different vaccine strains, and it is possible that neither of these represented the optimum cell-line for the re-isolation of the strain in question. As a result, it could be that some dogs shed virus which was not detected as a consequence of being less permissible on A72 or CRFK cells.

**Table 5:** Vaccine virus shedding.

Group (Vaccine)	Pup ID	Vaccinate/Sentinel	Days on Which Virus Shed
**1 (Nobivac DP PLUS)**	8638	V1	6, 7, 8, 9
	9012	S1	nd
	8668	V2	8, 9, 10
	8644	S2	11, 15, 16, 17
	9040	V3	9, 10, 11
	2681	S3	13, 14, 15
	8662	V4	6, 7
	8654	S4	11, 12, 13, 14
	8728	V5	6, 7
	8648	S5	6, 10, 11, 12
**2 (Vanguard Plus 5)**	1848	V1	nd
	1990	S1	nd
	9426	V2	nd
	2318	S2	nd
	9425	V3	nd
	1988	S3	nd
	1849	V4	nd
	9429	S4	nd
**3 (Versican Plus P)**	4602	V1	nd
	4541	S1	nd
	4603	V2	nd
	4596	S2	nd
	4546	V3	9, 10
	4542	S3	nd
	4545	V4	7, 8, 9, 10
	4599	S4	nd
**4 (Eurican Primo P)**	4601	V1	nd
	4597	S1	nd
	4547	V2	nd
	4595	S2	nd
	4544	V3	nd
	4548	S3	nd
	4543	V4	10, 11
	4598	S4	nd

nd = not detected.

## 4. Discussion

Dogs are naturally infected with canine parvovirus via oronasal exposure to contaminated faeces or fomites. Following exposure, virus replication begins in the lymphoid tissue of the oropharynx and the virus disseminates to the thymus, other lymphoid tissues and the intestinal crypts of the small intestine by means of a plasma viraemia and via infected lymphoid cells [12]. The virus replicates in the rapidly dividing lymphocytes of the thymus and in crypt cells at the base of villi in the small intestine. Viral replication and apoptosis cause immunosuppression through lymphocytolysis and compromise the gut barrier via destruction of the germinal epithelium of the small-intestinal crypts, shortening the villi and reducing their ability to assimilate nutrients and absorb water. This causes a severe enteritis with high morbidity and mortality, especially in young animals [12,13]. Live attenuated CPV vaccines are delivered parenterally, but nevertheless, the vaccine virus disseminates to the gut where virus replication occurs and virus is shed in the faeces [21]. In the presence of MDA vaccine virus may be neutralised, and if levels of MDA are sufficiently high complete neutralisation will occur and the vaccine virus will be unable to replicate.

Vaccination studies are required under European legislation to demonstrate safety and efficacy. If an additional claim for efficacy in the face of MDA is being made, then this too must be substantiated with animal studies [28]. These studies are almost always performed in groups of pups housed together in a single room. MDA studies with canine parvovirus present particular problems in that the virus, including vaccine virus, is robust and may persist in the environment, and vaccine virus may also be shed from vaccinated animals. The situation may then all too easily arise where, in a group of dogs, vaccination is only effective in a fraction of the group, the ones with lower levels of MDA, and these animals then shed vaccine virus in the faeces. The timepoint at which an individual dog begins to shed virus is generally correlated to the level of MDA present on the day of vaccination and thus variable (unpublished data). Subsequently, this shed vaccine virus can infect others of the group that were initially refractive to vaccination because of high MDA levels but have become susceptible due to a decline in their MDA levels over time, something which has previously been postulated [29]. Overall, this would give an inaccurate assessment of the ability of the vaccine to stimulate immunity in the presence of MDA.

The study design employed here demonstrates this potential problem in carrying out vaccine studies in group-housed dogs. Vaccine virus that is shed from one animal can be taken up by another and effective immunisation attributed to vaccination rather than secondary exposure. Although the group-housed situation may mirror what happens when litters of puppies are vaccinated at the breeders, it is important that we can accurately assess the response of the individual MDA-positive pup to a dose of vaccine since this will reflect the much more common situation where a non-immunised pup from a single household is presented for vaccination by its new owners.

It is evident from this study that some of the dogs vaccinated with the Vanguard Plus 5, Versican Plus P, and Eurican Primo P vaccines, despite having MDA titres at the time of vaccination of up to 288, were able to respond to vaccination, with the response occurring 7–14 days post vaccination. However, not all dogs responded, and those with titres >288 at the time of vaccination were unable to mount an immune response. Furthermore, it is interesting to note that vaccine virus shed from the Versican Plus P- and Eurican Primo P-vaccinated pups was unable to overcome the relatively low levels of MDA present at that time in the sentinel pups. The cell lines used in this study may not, however, have been the most optimal for all the vaccine strains [30], potentially leading to undetected virus shedding, which could underestimate virus transmission capacity. The CPV vaccine strain 630a used in Nobivac DP PLUS has previously been shown to overcome high levels of MDA [20] and here demonstrates its ability to overcome higher levels of MDA as compared to the other vaccines used.

Statistically speaking, it was not possible to undertake a conventional inferential analysis to model the trajectory of seroconversion in this study because different numbers of pups with different levels of MDA seroconverted on different days. We have therefore presented these data descriptively in Table 3, using grey shading to indicate seroconversion. It was, however, possible to use inferential statistical models to compare the antibody titres in the Nobivac DP PLUS group (including and excluding sentinels) with the titres in each of the other vaccine groups as a surrogate measure of seroconversion (as shown in Table 4). Both of these analyses demonstrate a significantly higher level of seroconversion in the Nobivac DP PLUS group compared to any of the other vaccine groups.

The superior efficacy of the CPV strain 630a in the presence of MDA is incompletely understood but is hypothesised by the authors to be linked to the method of attenuation. Live attenuated vaccine strains are classically generated using the Pasteurian approach of sequential passage in cell culture (or a different animal species) until genetic adaptations that enhance replication in the novel cell line lead to attenuated replication in the host animal [30]. In contrast to classically attenuated canine parvovirus vaccine strains, CPV strain 630a is a second-generation canine parvovirus vaccine that was constructed using recombinant DNA technology [20]. By inserting targeted attenuating amino acid substitutions into the receptor binding VP2 capsid gene of a field virus and placing this into a viral backbone with an attenuated non-structural gene, it was possible to sufficiently attenuate the virus without over attenuating it *in vivo*. Since the virus only replicates efficiently in rapidly dividing cells [12], we hypothesise that CPV 630a may have retained its ability to efficiently infect lymphocytes, which then carry the virus in an immune-privileged site hidden from the maternal antibodies. In this scenario, as the maternal antibodies decay vaccine-virus-infected lymphocytes will persist longer than the maternal antibody, thus allowing virus replication and subsequent seroconversion to occur.

## 5. Conclusions

In conclusion, different canine parvovirus vaccines differ in their ability to replicate in and immunise puppies in the presence of MDA, the levels of which may vary widely between puppies within or between litters and of different ages. The propensity for transmission of parvoviruses into the environment, together with the inquisitive nature of the dog, should be a consideration when designing studies to demonstrate efficacy that is applicable to the field situations in which the vaccines will ultimately be used. With MDA interference being a major cause of immunisation failure, and the importance of early socialisation becoming increasingly recognised, it is important that practitioners base their recommendations on the best available scientific understanding of the factors influencing vaccine efficacy in young puppies. This should ensure greater success in protecting dogs against the continuing threat of canine parvovirus.

## Figures and Tables

**Table 1:** Details of the vaccines used.

Vaccine	Company	Antigens and Titres (TCID_50_) per Dose *
**Nobivac DP PLUS**	MSD Animal Health	CPV strain 630a 10^5.1^ to 10^6.7^
		CDV strain Onderstepoort 10^5.1^ to 10^6.5^
**Vanguard Plus 5**	Zoetis	CPV strain NL-35-D ≥ 10^7.0^
		CDV strain N-CDV ≥ 10^3.0^
		CAV strain Manhattan ≥ 10^3.2^
		CPi strain NL-CPI-5 ≥ 10^6.0^
**Versican Plus P**	Zoetis	CPV strain Bio 12/B 10^4.3^ to 10^6.6^
**Eurican Primo P**	Boehringer Ingelheim	CPV strain 115-780916 ≥ 10^5.5^

* Information from the official product leaflets.

## Data Availability

The data presented in this study are available in this article and Supplementary Materials.

## Supplementary Materials

The following supporting information can be downloaded at: https://www.mdpi.com/article/10.3390/vaccines13080832/s1, Table S1: Serological response of the pups: haemagglutination inhibition (HAI units) against each of the three subtypes (2a, 2b, 2c); Table S2: Viral shedding: approximate viral load estimations (log_10_ TCID_50_/swab).
