# Supplementary material for: Assessing Canine Parvovirus Vaccine Performance in Puppies with Maternally Derived Antibody: An Improved Study Design
Source: Vaccines (Basel). 2025 Aug 4;13(8):832. doi: 10.3390/vaccines13080832 (PMC12390283; doi:10.3390/vaccines13080832)
Supplement: Supplementary file 1 [file vaccines-13-00832-s001.zip › vaccines-3662336-supplementary.pdf]

Table S1 (a). Serological Response of the Pups: Haemagglutination Inhibition titres (HAI units) against each of the three subtypes (2a, 2b, 2c).

Group	Pup ID	V/S*	Study Day																	
			0			3			5			7			9			11		
			2a	2b	2c	2a	2b	2c	2a	2b	2c	2a	2b	2c	2a	2b	2c	2a	2b	2c
1	**8638**	**V1**	416	576	576	288	416	416	144	208	288	52	72	72	9216	9216	9216	9216	13,312	13,312
	**9012**	**S1**	416	576	416	288	288	416	208	208	288	144	208	144	144	144	208	144	144	144
	**8668**	**V2**	288	576	416	288	416	416	144	208	208	52	104	104	416	1152	832	9216	9216	4608
	**8644**	**S2**	288	576	416	288	416	416	288	288	288	208	208	288	144	288	288	144	144	208
	**9040**	**V3**	288	416	576	288	416	298	288	288	288	144	144	144	144	144	144	18,432	13,312	8432
	**2681**	**S3**	104	104	144	72	104	104	52	72	72	36	36	36	52	36	36	<16	<16	<16
	**8662**	**V4**	104	144	144	72	104	72	36	36	36	832	832	1152	9216	13,312	7168	9216	9216	3328
	**8654**	**S4**	72	104	104	36	72	52	36	72	52	36	52	52	36	36	36	72	72	72
	**8728**	**V5**	72	72	72	72	72	72	26	36	26	832	1664	2304	13,312	13,312	9216	13,312	13,312	9216
	**8648**	**S5**	52	52	36	36	36	36	36	36	36	26	36	36	<16	<16	<16	6400	2304	1664
2	**1848**	**V1**	288	144	208	144	144	144	144	144	144	144	104	144	104	72	104	144	144	144
	**1990**	**S1**	144	72	144	104	72	104	104	72	104	72	72	72	72	52	52	72	52	72
	**9426**	**V2**	208	144	208	144	104	144	144	112	144	144	72	104	144	104	144	288	208	288
	**2318**	**S2**	104	72	104	72	52	72	72	72	72	52	36	52	36	36	36	36	36	36
	**9425**	**V3**	208	104	208	104	72	104	144	72	104	288	144	288	1664	1664	2304	2304	3328	3328
	**1988**	**S3**	104	52	104	72	72	72	72	72	72	52	36	72	36	36	36	36	26	36
	**1849**	**V4**	72	72	72	72	52	52	72	72	72	288	288	288	832	832	1152	4608	4608	4608
	**9429**	**S4**	72	36	72	72	36	52	52	36	36	36	26	26	36	26	28	36	26	36

* V = Vaccinate; S = Sentinel, Grey shading: Active seroconversion. The start of the grey shading is the first indication of an active immune response to vaccination.

Table S1 (a) (cont.). Serological Response of the Pups: Haemagglutination Inhibition titres (HAI units) against each of the three subtypes (2a, 2b, 2c).

Group	Pup ID	V/S*	Study Day														
			14			18			21			25			28		
			2a	2b	2c	2a	2b	2c	2a	2b	2c	2a	2b	2c	2a	2b	2c
1	8638	V1	18,432	32,768	20,480	18,432	18,432	32,768	14,336	28,672	18,432	9216	13,312	13,312	13,312	9216	13,312
	9012	S1	144	144	144	104	144	104	144	144	144	72	144	104	144	72	72
	8668	V2	13,312	13,312	13,312	9216	13,312	13,312	9216	14,336	14,336	13,312	18,432	18,432	14,336	13,312	18,432
	8644	S2	18	18	16	9216	18,432	13,312	18,432	28,672	28,672	13,312	28,672	18,432	9216	6656	13,312
	9040	V3	>40,960	>40,960	>40,960	>40,960	>40,960	>40,960	>81,920	>81,920	>81,920	57,344	36,864	40,960	≥40,960	≥40,960	≥40,960
	2681	S3	104	72	112	9216	7168	13312	9216	14,336	9216	5120	9216	6656	3328	3328	6656
	8662	V4	18,432	13,312	18,432	18,432	18,432	18,432	9216	14,336	14,336	14,336	18,432	18,432	6656	9216	13,312
	8654	S4	6656	9216	9216	18,432	14,336	32,768	14,336	28,672	18,432	9216	18,432	14,336	9216	6656	13,312
	8728	V5	18,432	32,768	10,752	18,432	18,432	18,432	9216	18,432	14,336	14,336	18,432	18,432	9216	9216	13,312
	8648	S5	18,432	18,432	13,312	18,432	18,432	18,432	18,432	18,432	14,336	9216	9216	9216	6656	6656	9216
2	1848	V1	144	104	104	52	72	72	52	36	36	36	36	36	36	18	36
	1990	S1	72	52	52	36	36	36	36	18	36	36	18	28	18	18	18
	9426	V2	144	144	144	72	72	72	72	52	52	52	36	36	36	36	36
	2318	S2	36	36	36	26	18	36	26	18	26	26	18	28	<16	<16	16
	9425	V3	1664	1664	2304	1152	1152	1152	832	832	1152	576	832	576	576	576	832
	1988	S3	36	36	36	26	18	36	26	18	26	26	18	18	16	<16	18
	1849	V4	4608	3328	3328	2304	2304	3328	1664	2304	2304	1664	1664	2304	1664	1664	3328
	9429	S4	36	18	18	18	18	18	18	18	18	18	18	18	16	<16	<16

* V = Vaccinate; S = Sentinel, Grey shading: Active seroconversion. The start of the grey shading is the first indication of an active immune response to vaccination.

Table S1 (b). Serological Response of the Pups: Haemagglutination Inhibition titres (HAI units) against each of the three subtypes (2a, 2b, 2c).

Group	Pup ID	V/S*	Study Day 0			3			5			7			9			11		
			**2a**	**2b**	**2c**	**2a**	**2b**	**2c**	**2a**	**2b**	**2c**	**2a**	**2b**	**2c**	**2a**	**2b**	**2c**	**2a**	**2b**	**2c**
**3**	**4602**	**V1**	576	288	576	288	144	288	288	144	144	208	144	144	208	144	144	288	104	144
	**4541**	**S1**	288	288	288	208	144	144	144	144	144	208	144	144	144	104	144	144	104	144
	**4603**	**V2**	448	288	448	288	144	208	288	144	144	208	72	144	208	104	144	144	72	144
	**4596**	**S2**	144	104	144	104	72	72	72	72	72	104	56	72	72	52	72	72	36	52
	**4546**	**V3**	288	288	288	288	144	288	288	144	288	288	144	224	72	72	72	576	288	576
	**4542**	**S3**	288	288	288	288	144	288	288	144	288	208	144	144	208	144	208	144	104	144
	**4545**	**V4**	144	104	144	104	72	72	104	72	72	104	72	72	288	288	288	13312	6656	7168
	**4599**	**S4**	144	104	144	72	52	72	72	52	52	72	36	52	72	36	72	52	36	52
**4**	**4601**	**V1**	576	288	576	288	144	288	288	208	192	288	144	168	288	144	224	208	104	144
	**4597**	**S1**	288	208	288	144	104	144	144	104	112	144	72	104	144	72	144	104	52	72
	**4547**	**V2**	416	288	416	288	144	288	288	208	288	144	144	104	208	144	224	144	104	144
	**4595**	**S2**	288	208	288	144	104	144	144	104	144	144	72	104	144	72	144	144	72	104
	**4544**	**V3**	288	208	288	208	144	208	208	144	144	144	144	144	144	104	144	144	72	144
	**4548**	**S3**	144	104	144	104	52	72	104	72	72	52	56	72	72	36	52	52	36	52
	**4543**	**V4**	144	104	144	104	52	72	72	72	72	52	72	72	72	72	72	832	576	1152
	**4598**	**S4**	104	104	104	72	36	72	72	36	52	72	56	72	52	26	36	36	18	36

* V = Vaccinate; S = Sentinel, Grey shading: Active seroconversion. The start of the grey shading is the first indication of an active immune response to vaccination.

Table S1 (b) (cont.). Serological Response of the Pups: Haemagglutination Inhibition titres (HAI units) against each of the three subtypes (2a, 2b, 2c).

Group	Pup ID	V/S*	Study Day 14			18			21			25			28		
			2a	2b	2c	2a	2b	2c	2a	2b	2c	2a	2b	2c	2a	2b	2c
3	**4602**	**V1**	208	144	144	144	72	104	144	72	144	72	36	72	72	36	52
	**4541**	**S1**	144	72	104	112	72	72	72	52	112	72	36	52	72	36	52
	**4603**	**V2**	144	72	72	104	72	72	72	36	72	72	36	36	72	26	36
	**4596**	**S2**	72	36	52	36	36	36	36	18	36	18	18	18	36	8	18
	**4546**	**V3**	9216	18432	9216	13312	13312	13312	6656	6656	14336	6656	9216	6656	9216	7168	9216
	**4542**	**S3**	208	144	144	144	72	72	104	72	144	72	36	72	72	36	52
	**4545**	**V4**	36864	36864	26624	36864	36864	26624	18432	28672	36864	26624	18432	18432	18432	18432	18432
	**4599**	**S4**	72	56	52	36	26	36	36	18	36	18	18	18	26	14	18
4	**4601**	**V1**	144	104	144	144	72	104	144	72	104	72	52	72	72	36	72
	**4597**	**S1**	104	72	72	52	52	52	36	36	52	36	26	36	52	18	36
	**4547**	**V2**	144	104	144	144	56	104	72	72	104	72	52	72	72	36	72
	**4595**	**S2**	104	72	72	72	52	72	72	36	72	36	26	36	52	36	36
	**4544**	**V3**	832	576	832	3584	3328	2304	2304	2304	3328	1664	1664	1152	1152	1152	1152
	**4548**	**S3**	72	36	36	36	26	36	36	18	36	18	18	18	18	14	18
	**4543**	**V4**	13312	18432	9216	9216	10752	9216	4608	6656	9216	3328	4608	4608	3328	4608	4608
	**4598**	**S4**	72	36	36	36	26	36	36	18	36	18	18	18	18	18	18

* V = Vaccinate; S = Sentinel, Grey shading: Active seroconversion. The start of the grey shading is the first indication of an active immune response to vaccination.

Table S2. Viral Shedding: Approximate Viral Load Estimations (log_{10} $TCID_{50}$/swab).

Group	Pup ID	V/S*	Study Day																
			0-4	5	6	7	8	9	10	11	12	13	14	15	16	17	18	19	20-28
1	8638	V1	-	-	3.15	3.84	2.1	2.8	-	-	-	-	-	-	-	-	-	-	-
	9012	S1	-	-	-	-	-	-	-	-	-	-	-	-	-		-	-	-
	8668	V2	-	-	-	-	2.45	3.49	4.19	-	-	-	-	-	-	-	-	-	-
	8644	S2	-	-	-	-	-	-	-	2.45	-	-	-	>2.80 ‡	3.15	3.84	-	-	-
	9040	V3	-	-	-	-	-	3.49	3.84	1.4	-	-	-	-	-	-	-	-	-
	2681	S3	-	-	-	-	-	-	-	-	-	3.15	2.1	5.94	-	-	-	-	-
	8662	V4	-	-	2.8	3.49	-	-	-	-	-	-	-	-	-	-	-	-	-
	8654	S4	-	-	-	-	-	-	-	3.15	4.19	3.32	3.84	-	-	-	-	-	-
	8728	V5	-	-	4.19	3.49	-	-	-	-	-	-	-	-	-	-	-	-	-
	8648	S5	-	-	2.45	-	-	-	2.1	5.94	3.49	-	-	-	-	-	-	-	-
2	1848	V1	-	-	-	-	-	-	-	-	-	-	-	-	-	-	-	-	-
	1990	S1	-	-	-	-	-	-	-	-	-	-	-	-	-	-	-	-	-
	9426	V2	-	-	-	-	-	-	-	-	-	-	-	-	-	-	-	-	-
	2318	S2	-	-	-	-	-	-	-	-	-	-	-	-	-	-	-	-	-
	9425	V3	-	-	-	-	-	-	-	-	-	-	-	-	-	-	-	-	-
	1988	S3	-	-	-	-	-	-	-	-	-	-	-	-	-	-	-	-	-
	1849	V4	-	-	-	-	-	-	-	-	-	-	-	-	-	-	-	-	-
	9429	S4	-	-	-	-	-	-	-	-	-	-	-	-	-	-	-	-	-

* V = Vaccinate; S = Sentinel, (-) Rectal swab negative for canine parvovirus, ‡ 2.80 to 3.15; one well of the two wells not readable

Table S2 (cont.). Viral Shedding: Approximate Viral Load Estimations (log_{10} $TCID_{50}$/swab).

Group	Pup ID	V/S*	Study Day																
			**0-4**	**5**	**6**	**7**	**8**	**9**	**10**	**11**	**12**	**13**	**14**	**15**	**16**	**17**	**18**	**19**	**20-28**
**3**	**4602**	**V1**	-	-	-	-	-	-	-	-	-	-	-	-	-	-	-	-	-
	**4541**	**S1**	-	-	-	-	-	-	-	-	-	-	-	-	-	-	-	-	-
	**4603**	**V2**	-	-	-	-	-	-	-	-	-	-	-	-	-	-	-	-	-
	**4596**	**S2**	-	-	-	-	-	-	-	-	-	-	-	-	-	-	-	-	-
	**4546**	**V3**	-	-	-	-	-	3.15 ^	2.80 ^	-	-	-	-	-	-	-	-	-	-
	**4542**	**S3**	-	-	-	-	-	-	-	-	-	-	-	-	-	-	-	-	-
	**4545**	**V4**	-	-	-	≥2.10 ‡	2.45	2.10	2.10 ^	-	-	-	-	-	-	-	-	-	-
	**4599**	**S4**	-	-	-	-	-	-	-	-	-	-	-	-	-	-	-	-	-
**4**	**4601**	**V1**	-	-	-	-	-	-	-	-	-	-	-	-	-	-	-	-	-
	**4597**	**S1**	-	-	-	-	-	-	-	-	-	-	-	-	-	-	-	-	-
	**4547**	**V2**	-	-	-	-	-	-	-	-	-	-	-	-	-	-	-	-	-
	**4595**	**S2**	-	-	-	-	-	-	-	-	-	-	-	-	-	-	-	-	-
	**4544**	**V3**	-	-	-	-	-	-	-	-	-	-	-	-	-	-	-	-	-
	**4548**	**S3**	-	-	-	-	-	-	-	-	-	-	-	-	-	-	-	-	-
	**4543**	**V4**	-	-	-	-	-	-	≤1.22 #	2.10	-	-	-	-	-	-	-	-	-
	**4598**	**S4**	-	-	-	-	-	-	-	-	-	-	-	-	-	-	-	-	-

* V = Vaccinate; S = Sentinel, (-) Rectal swab negative for canine parvovirus, ^ Some lower dilutions not readable but positive wells in higher dilutions, ‡ One well of the two wells not readable in 1:25 (second) dilution, other well positive, # Dilution 1:5 two wells negative, dilution 1:25 one of two wells positive
